# The Effect of Pediatric Liver Transplantation on Depression Levels in Children and the Potential Role of Liver Enzymes as Biomarkers

**DOI:** 10.3390/medicina62061148

**Published:** 2026-06-12

**Authors:** Serkan Suren, Deniz Yavuz Baskiran, Irem Tulum, Adil Baskiran, Sezai Yilmaz

**Affiliations:** 1 Child and Adolescent Psychiatry Practice, Samsun 55200, Türkiye; drserkansuren@hotmail.com (S.S.); iremttulum@gmail.com (I.T.); 2 Liver Transplantation Institute, Inonu University, Malatya 44000, Türkiye; dr.adil.baskiran@gmail.com (A.B.); sezai.yilmaz@inonu.edu.tr (S.Y.)

**Keywords:** liver transplantation, depression, post-traumatic stress disorders, liver function tests, biomarkers

## Abstract

*Background and Objectives*: This study aimed to examine the level of depression in children who had undergone pediatric liver transplantation and to evaluate the potential role of liver enzymes as biomarkers of depression. *Materials and Methods*: The study was conducted with 50 pediatric liver transplant recipients followed at the Liver Transplantation Institute of İnönü University, and data were collected through face-to-face interviews. The Personal and Transplant Information Form, Child Revised Impact of Event Scale, and Patient Health Questionnaire–Depression were used as data collection tools. In addition to descriptive statistics, Student’s *t*-test, Mann–Whitney U test, correlation analyses, and regression analyses were performed. *Results*: The median PHQ-9 score was 1.00 (Q1–Q3: 0.00–5.00), indicating generally low levels of depression. A significant positive correlation was found between CRIES and PHQ-9 scores (r = 0.414, *p* < 0.01). In contrast, no consistent significant associations were observed between liver enzyme levels and depression scores in multivariate analyses, although bilirubin showed a modest negative correlation with PHQ-9 scores. In the multivariate analysis, although the overall regression model was not statistically significant, the CRIES score showed an individual association with PHQ-9 scores within the model (B = 0.117, *p* = 0.037). *Conclusions*: Liver enzymes cannot be considered strong biomarkers of depression in pediatric liver transplant recipients; however, post-traumatic stress symptoms may be an important clinical indicator for assessing psychological adjustment.

## 1. Introduction

Solid organ transplantation has become one of the fundamental treatment methods that prolongs life and significantly improves prognosis in individuals with end-stage organ failure [1,2,3]. Advances in diagnosis, surgical techniques, and postoperative care in recent years have contributed to a significant increase in survival rates, particularly among pediatric organ transplant recipients. Five-year survival rates are reported to be approximately 70% after lung transplantation, 75–90% after heart and liver transplantation (LT), and over 95% after kidney transplantation [4]. According to current data, as of March 2024, 2151 children and adolescents worldwide were on the organ transplant waiting list [5]. In the United States, 9528 LTs were performed in 2022, and this number had reached 6143 since the beginning of 2023 [6]. In Türkiye, although there has been an increase in LT procedures, official data detailing this increase are limited [7]. Moreover, long-term outcomes have improved significantly, with many patients achieving survival beyond 10 years in both adult and pediatric populations. Recent registry data indicate that post-transplant mortality continues to decrease over time, reflecting improvements in surgical techniques and long-term management [8].

Children and adolescents diagnosed with end-stage organ failure face numerous medical and psychological stressors throughout the course of their illness [9,10,11]. Invasive diagnostic procedures, repeated medical interventions, surgical procedures, life-threatening situations, and intensive care experiences are among the potentially traumatic experiences in this age group [9]. Despite increased survival rates, pediatric organ transplantation also involves complex clinical processes such as the need for long-term immunosuppressive treatment, frequent hospitalizations, multiple surgical procedures, and problems related to growth and nutrition. Furthermore, organ transplantation is considered a life experience involving significant psychosocial difficulties for pediatric and adolescent patients [12]. Physical changes experienced by children during the post-transplant process can also affect their body image perception. These physical changes that occur during the transplant process can negatively affect children’s self-confidence levels. Weight gain, short stature, and other changes in appearance can lead to a distorted body image, while negative feedback from peers can make it difficult to establish and maintain social relationships [13]. Therefore, this situation can have an impact on mental health. Furthermore, psychosocial factors such as psychiatric comorbidities, anxiety symptoms, limited social support, a history of substance use, and lack of adequate caregiver support have been reported to be associated with reduced adherence to post-transplant treatment [10]. Therefore, identifying and addressing psychosocial risks in pediatric organ transplant recipients is crucial for both improving individual well-being and enhancing long-term transplant outcomes [14]. However, some studies suggest that although children’s physical functioning is lower than that of healthy peers in the post-transplant period, their self-esteem and overall mental health levels can be maintained [15].

Although the assessment of mood changes in the post-transplant period is mostly based on self-reports, biological markers may play a complementary role in this process [16,17]. Liver dysfunction may be associated with a range of physical symptoms such as fatigue, pruritus, abdominal discomfort, and jaundice, which can negatively affect quality of life and psychological well-being. In addition, alterations in liver function may influence neurobiological processes through mechanisms such as systemic inflammation and metabolic changes, potentially contributing to the development of depressive symptoms [16,18]. A previous study found significant associations between depression and chronic liver disease subgroups such as metabolic-related fatty liver disease, alcohol-related liver disease, viral hepatitis, and drug-induced liver injury [16]. Current research has revealed statistically significant associations between depressive symptoms and liver enzymes such as γ-glutamyl transferase (GGT) [19]. Furthermore, it has been suggested that certain enzyme ratios, such as the AST/ALT ratio, may be associated with emotional symptom severity; however, it remains unclear whether these changes reflect physiological processes related to liver function or indirect associations with psychological status [18,19]. Considering all influencing factors, a detailed assessment of the mental health needs of pediatric organ transplant recipients is particularly important because mental health issues in this age group often progress unnoticed and frequently emerge only during functional decline or crises [11]. However, the majority of the existing literature has focused on examining hepatic or renal dysfunction in adults diagnosed with depression; pediatric organ transplant recipients have been relatively neglected in this regard [20]. Considering this gap in the literature, this study aimed to examine depression levels in children after a major medical event such as pediatric LT and to evaluate the potential role of liver enzymes as biomarkers of depressive symptoms. This study, which addresses the relationship between psychological symptoms and biochemical liver indicators in pediatric liver transplant recipients, is unique in that it provides a new and comprehensive contribution to the limited literature on the use of objective biological markers in depression assessments. Furthermore, this study focused solely on children who had a smooth follow-up period after liver transplantation to observe the effects of the transplantation event on mental health. In the present study, commonly used liver function parameters were selected as potential biomarkers due to their clinical relevance in the routine follow-up of pediatric liver transplant recipients. By excluding confounding factors, the study evaluated the effects of transplantation surgery and its aftermath on depression. In this respect, the study also makes important contributions to the literature.

### Research Questions

- What is the level of depressive symptoms in children who did not experience complications after pediatric liver transplantation?
- Is there a relationship between post-traumatic stress levels and depressive symptoms in these children?
- Do depressive symptoms differ according to sociodemographic and transplantation-related characteristics?

## 2. Materials and Methods

### 2.1. Research Design

This study was conducted in a cross-sectional design to determine the effect of pediatric LT on depression levels in children and the potential role of liver enzymes as biomarkers. A cross-sectional design was considered appropriate for this study as it allows for the evaluation of relationships between psychological and biochemical variables at a single point in time in a clinically stable population.

### 2.2. Research Population

The study was conducted at the Turgut Ozal Medical Center Liver Institute between January 2026 and February 2026. The study population consisted of children aged 4–18 years who had undergone liver transplantation at this institute. A total of 93 pediatric liver transplants performed in this unit over the last three years (2023–2024–2025) was accepted as the population size. Since the population size was accessible, no separate sampling method was applied, and the aim was to reach the entire population. A census sampling approach was used as the aim was to include all eligible patients within the defined population. Since the study aimed to include the entire accessible population, a formal sample size calculation was not performed. Of the population, 87 patients who met the inclusion criteria were reached. However, 37 patients and their families declined to participate, and the 50 pediatric patients who agreed to participate constituted the study sample. Children aged 4–18 years who had undergone liver transplantation at least six months prior to the study were included. Exclusion criteria included children with communication difficulties, those who had experienced post-transplant complications or rejection, those with repeated hospitalizations (defined as additional unplanned hospital admissions related to transplant complications), and those receiving psychiatric treatment.

### 2.3. Variables of the Study

The level of depression, measured by the PHQ-9 total score, was considered the dependent variable of this study. Independent variables included sociodemographic characteristics of the children and their parents (gender, educational status, presence of chronic illness in the family, and parental education levels), transplant-related characteristics (type of transplantation, age at transplantation, type of immunosuppressive medication, and presence of additional diseases), body mass index, liver enzyme levels (AST, ALT, GGT, and bilirubin), and post-traumatic stress levels measured by the CRIES. The primary outcome of this study was the level of depression measured by the PHQ-9 total score. Secondary outcomes included post-traumatic stress levels measured by the CRIES and biochemical parameters (AST, ALT, GGT, and bilirubin), as well as body mass index (BMI). The PHQ-9 score was considered the dependent variable, while sociodemographic and transplant-related characteristics were treated as independent variables.

### 2.4. Data Collection Tools

Data were collected using the “Personal and Transplant Information Form” created by the researchers, the “Child Revised Impact of Event Scale (CRIES)” with established language validity, and the “Patient Health Questionnaire-9”. A meter and a scale, calibrated annually and located in the outpatient follow-up clinic, were used to determine the body mass index. Registered laboratory results were used to monitor blood values.

Personal and Transplant Information Form: Developed by the researchers through a literature review [7,10,11]. As this form includes descriptive and factual variables, no validity or reliability analysis was performed. This form consists of nine closed-ended questions inquiring about the gender, age, parents’ age and education level, region of residence, presence of additional diseases, presence of chronic diseases in the family, type of transplant, and immunosuppressive medications of children who have undergone transplantation. The form also includes a section for recording height and weight measurements, as well as liver enzyme results (AST, ALT, GGT, and bilirubin) obtained from hospital records.

Child Revised Impact of Event Scale (CRIES): This scale, developed by the Children and War Foundation, determines children’s post-traumatic stress levels (PTSD) after an event and has been used in many countries for many years. The Turkish language validity and reliability study of the scale was conducted by Çeri and colleagues in 2021 [21]. The first eight items of the scale form the Intrusive Thoughts and Avoidance subscale, while the next five items form the Arousal subscale. The total score on the scale ranges from 0 to 65, with higher scores indicating greater PTSD. A cutoff score of 30 on the CRIES-13 and 17 on the CRIES-8 has been reported to provide maximum sensitivity and specificity in detecting PTSD [22]. While the internal consistency Cronbach’s alpha value of the original scale is 0.85, the Cronbach’s alpha value in this study was determined to be 0.88 [21]. A Cronbach’s alpha value above 0.70 is generally considered acceptable, indicating good internal consistency [23].

Patient Health Questionnaire–Depression (PHQ-9): This scale, which assesses 9 symptoms of depression according to DSM-IV criteria, is a diagnostic tool used to identify common mental disorders such as depression. The scale was developed by Kroenke and colleagues [24], and its Turkish validity and reliability study was conducted by Sari and colleagues in 2016 [25]. According to the original questionnaire’s scoring system, scores between 1 and 4 are considered minimal, 5 to 9 mild, 10 to 14 moderate, 15 to 19 moderately severe, and 20 to 27 severe depression. While the internal consistency Cronbach’s alpha value of the original scale was 0.84 [25], the Cronbach’s alpha value in this study was determined to be 0.87. A Cronbach’s alpha value above 0.70 is generally considered acceptable, indicating good internal consistency [23].

### 2.5. Data Collection

The data for the study were collected in the outpatient clinic environment at the Turgut Ozal Medical Center Liver Transplant Institute between January and February 2026 from children aged 4–18 who had undergone transplantation and were being followed up on an outpatient basis. The details of the study were explained to the parents of children who agreed to participate in the study during a telephone interview, and an appointment schedule was arranged with the parents who agreed to participate in the study, according to the date of their follow-up visit. Patients who came to the outpatient clinic were asked to fill out the data collection tools accompanied by their parents. For children who could not read or write, the form items were read by the researcher. For younger children who were unable to adequately read or comprehend the questionnaire items, the forms were completed with parental assistance and researcher guidance. The PHQ-9 was used as a brief screening tool rather than a diagnostic instrument. Children who did not have reading and comprehension problems filled out the data collection tools themselves. It took approximately 15–20 min to complete the forms for each patient. A meter and scale available at the outpatient clinic were used to assess the children’s height and weight, and each child’s height and weight were measured by the researchers. The children’s liver enzymes were assessed as part of routine follow-up. Blood test results evaluated on the day of their visit for control were included in the evaluation. Planning was done so that the date of liver enzyme evaluation coincided with the date on which the forms were filled out.

### 2.6. Ethical Statement of the Research

Before the research data were collected, ethical approval was obtained from the Inonu University Non-Interventional Clinical Research Ethics Committee (Decision No: 2026/9280). After this approval, institutional permission was obtained from the center where the research was conducted. When determining the group of patients to be included in the study, telephone interviews were first conducted with the parents of children who had undergone LT. Information about the study was provided, and verbal consent was obtained. During the data collection phase, written consent was obtained from the parents and verbal consent from children. Ethical principles were adhered to at every stage of the study, and the study was conducted in accordance with the Declaration of Helsinki.

### 2.7. Statistical Analysis of the Research

The statistical analysis of the research data was performed using SPSS 25 (IBM Corp. Released 2017. IBM SPSS Statistics for Windows, Version 25.0. Armonk, NY, USA: IBM Corp.) software. As the sample size in this study was 50, the Kolmogorov–Smirnov test was used to assess the normality of the data [26]. Skewness and kurtosis values were considered for normal distribution, and variable distributions between +2 and −2 were considered normal. Descriptive statistics (mean, standard deviation, median, minimum, maximum, number, and percentile) were provided for categorical and continuous variables in the study. The Student’s *t*-test was used to compare categorical variables with continuous variables. The relationship between two continuous variables was evaluated using the Pearson correlation coefficient, and when the parametric test prerequisites were not met, the Spearman correlation coefficient was used. One of the techniques used to determine the relationship between variables was regression analysis. In regression analysis, the dependent and independent variables must be continuous variables measured on at least an interval scale and must be normally distributed [27]. Based on this, regression analysis was used in our study to evaluate the effect of independent variables on various parameters. Potential confounding variables, including sociodemographic characteristics and transplant-related factors, were included in the regression analysis to control for their effects on depression scores. A *p*-value < 0.05 was considered statistically significant.

## 3. Results

The statistical analysis results of the data obtained from a total of 50 pediatric transplant patients participating in the study are presented.

The results of the statistical analysis conducted on 50 pediatric transplant recipients are presented in Table 1. The median age of the participants was 13.50 years (Q1–Q3: 11.00–15.50), and the median age at transplantation was 66 months (Q1–Q3: 36.00–108.00). The median maternal and paternal ages were 38.50 years (Q1–Q3: 34.00–42.00) and 42.00 years (Q1–Q3: 38.00–46.00), respectively. The median CRIES score of the children was 8.00 (Q1–Q3: 2.00–18.00), while the median PHQ-9 score was 1.00 (Q1–Q3: 0.00–5.00), indicating generally low levels of post-traumatic stress and depressive symptoms. Due to the non-normal distribution of the biochemical variables, enzyme levels were expressed as medians (Q1–Q3). The median values of AST, ALT, bilirubin, and GGT were 32.00 (24.75–58.50) U/L, 27.00 (16.00–71.00) U/L, 0.59 (0.42–0.89) mg/dL, and 27.50 (17.00–93.50) U/L, respectively. The median BMI was 19.10 (16.55–21.73) kg/m^2^.

According to the socio-demographic characteristics of the participants, 52% were male, 82% were continuing their education, 80% had no chronic disease in their family, 88% had received a transplant from a living donor, 86% used immunosuppressive drugs with the active ingredient tacrolimus, 98% had no additional disease, 48% had mothers with a primary school education, and 60% had fathers with a primary school education. When the differences in depression levels were evaluated according to these variables, no significant differences were found among the variables (*p* > 0.05). However, the mean depression score was 5.14 ± 5.08 (Md: 5.00), participants with additional diseases had 9.00 ± 0.00 (Md: 9.00), and those whose fathers had a high school education level had 5.87 ± 4.85 (Md: 7.00) means, indicating mild depression (Table 2).

The relationship between the CRIES score in transplanted children, age-related variables, liver enzymes, and the PHQ-9 total mean score was evaluated using Pearson Correlation analysis. At the end of the evaluation, a negative, mildly significant relationship was found between the PHQ-9 total mean and the bilirubin value (r = −0.310; *p* < 0.05), and a positive, moderately significant relationship between the CRIES total mean and the PHQ-9 total mean (r = 0.414; *p* < 0.01), (Table 3).

When the effects of liver enzymes and the CRIES total score on the PHQ-9 total score in transplanted children was evaluated using multivariate linear regression analysis, it was determined that the CRIES total score showed an individual association with the PHQ-9 total score (B = 0.117; *p* = 0.037). The independent variables explained 14.4% of the variance in depression scores (R^2^ = 0.144). When all variables were considered together, the model did not significantly explain the PHQ-9 scores (F = 1.482; *p* = 0.215), (Table 4).

## 4. Discussion

Patients undergoing transplantation face numerous physical, psychological, and social difficulties and sources of stress throughout the process, from diagnosis to treatment and follow-up [28]. These challenges may also include mental disorders such as depression and anxiety, which can negatively affect disease outcomes. A review focusing on post-transplant depression indicated that post-transplant depression is associated with increased mortality rates and the need for repeated healthcare services [29]. This study examined the relationship between psychological symptoms and biochemical liver markers in pediatric LT recipients. The study found that participants showed minimal signs of depression and, according to the scale used to determine the effect of transplantation on children’s stress levels, had low PTSD levels. Multidisciplinary care is essential during the transplant process, as mental disorders and treatment non-adherence may negatively affect outcomes [30]. Although post-traumatic stress levels were found to be relatively low in this study, previous studies have reported higher psychiatric symptom levels in transplant patients. For example, Uyar’s study reported higher depression, stress, and anxiety levels among kidney transplant recipients experiencing multiple side effects [31]. Similarly, depression and anxiety disorders have been reported in approximately one-third of heart transplant patients, particularly among those with comorbidities [32]. Differences between studies may be explained by variations in sample characteristics, particularly the inclusion of clinically stable patients without post-transplant complications in the present study. The inclusion of children who did not experience side effects, had no history of infection, and did not require hospitalization in this study may have contributed to the detection of lower levels of psychological symptoms. It should also be noted that a large proportion of the participants received transplants from living donors. Living donor transplantation is generally associated with shorter waiting times and better clinical outcomes, which may have contributed to the relatively low levels of depression observed in this study. This suggests that reduced physical complications may have decreased the psychological burden. When comparing depression levels based on the socio-demographic characteristics and transplant-related characteristics of the patients included in the study, no significant differences were found between the averages. However, relatively higher median depression scores were observed among participants using everolimus, those with additional diseases, and those whose fathers had a high school education level. Immunosuppressive treatment suppresses the immune system, making organ recipients more vulnerable to infections, which can lead to long-term social isolation. Restricted social interaction and fear of infection are important factors that can have negative effects on mental health [32]. The higher depression scores observed in patients using everolimus may be related to differences in drug profiles and clinical indications. Since everolimus is often used in more complex clinical cases, this may reflect a higher baseline clinical burden rather than a direct effect of the drug. However, this finding does not indicate causality and requires confirmation in larger studies. A previous study reported a bidirectional relationship between chronic diseases and mental health and that the burden of physical illness can increase psychological symptoms [33]. Unhealthy lifestyle behaviors and non-adherence to prescribed treatments can accelerate the progression of underlying diseases, further increasing the physical disease burden; this situation may interact with mental symptoms such as depression and anxiety [34]. In this context, recognizing mental health symptoms early and addressing them with a holistic approach can play an important role in preventing the disease course from being negatively affected.

Mental health symptoms are commonly reported after LT [35]. In this study, bilirubin levels showed a negative correlation with depression, while PTSD levels showed a positive correlation. Although the CRIES score showed a statistically significant individual coefficient in the regression analysis, the overall regression model was not statistically significant. Therefore, this finding should be interpreted cautiously and may reflect the limited sample size and exploratory nature of the analysis. Previous studies have also reported higher depression and anxiety levels in pediatric LT patients [36] and a positive association between PTSD and depression [37]. Differences between studies may be related to factors such as intensive care experience or post-transplant complications, which were not present in the current sample. A negative correlation was observed between bilirubin levels and PHQ-9 scores; however, this finding should be interpreted cautiously. Considering the relatively small sample size and the multiple comparisons performed in the study, the observed association may reflect a chance finding (type I error). In addition, the inclusion of only clinically stable patients without post-transplant complications may have introduced selection bias, which could have influenced the biochemical and psychological profiles of the participants. Therefore, further studies with larger and more heterogeneous samples are needed to clarify the relationship between bilirubin levels and depressive symptoms. Although there are studies in the literature reporting that depressive symptoms in kidney transplant patients and individuals with chronic kidney disease are similar [38], some studies suggest that the transplant process may positively affect the mental well-being of kidney transplant recipients [39,40]. Similar to the findings of this study, a study conducted with 56 young people who had undergone kidney, liver, or heart transplantation reported that the majority of participants did not show clinically significant depressive symptoms and that their average depression and anxiety levels were below those expected in the general population. The same study emphasized that 22.6% of young people scored above clinical thresholds according to trauma measurements and that positive developments in the postoperative period could have a protective effect on mental health. Similarly, the current study also found low levels of depression; however, it was determined that post-traumatic stress symptoms had a significant effect on depression and that depression symptoms increased as the level of trauma increased. These findings indicate that even if the overall level of depression is low, trauma symptoms may play a decisive role in mental adjustment. Although a negative correlation was observed between bilirubin levels and PHQ-9 scores, no consistent significant relationships were identified between the evaluated liver enzymes and psychological symptoms in the multivariate analyses. This finding is consistent with a limited number of studies evaluating the direct relationship between liver function tests and psychiatric symptoms, which similarly failed to show a clear link between biochemical markers and psychological symptoms [18]. The relationship between liver function and psychological status is likely complex and cannot be explained solely by routine enzyme levels.

From a clinical perspective, these findings highlight the importance of routine psychological screening in pediatric liver transplant recipients, even in clinically stable cases. The significant association between post-traumatic stress symptoms and depression suggests that trauma-focused assessments may be more informative than relying solely on biological markers. Early identification of at-risk children through simple psychological tools such as CRIES and PHQ-9 may contribute to timely psychosocial interventions and improved long-term outcomes. Although several potential confounding variables were considered in the analysis, it is possible that unmeasured factors may have influenced the observed relationships.

### Limitations

This study has some limitations. First, the cross-sectional design of the study does not allow for the interpretation of findings based on causal relationships. The fact that the sample consisted of clinically stable children with no history of side effects, infections, or hospitalizations may limit the generalizability of the findings to all transplant recipients. In addition, approximately 43% of eligible patients and their families declined participation, which may have introduced selection bias. Families experiencing greater psychological burden may have been less willing to participate, potentially contributing to the relatively low depression and post-traumatic stress scores observed in the study. Furthermore, since the mental health assessments used in the study were based on self-report scales, the possibility that responses were influenced by social desirability bias or perceptual differences cannot be ignored. Another limitation is that the study included younger children, including participants aged 4 years, for whom the PHQ-9 has limited validation evidence. Although parental assistance and researcher guidance were provided during data collection, the interpretation of depression symptoms in very young children should be approached cautiously. The use of single-time point measurements in assessing the relationship between biochemical parameters and mental health symptoms may have been insufficient to reflect temporal changes in these variables. Finally, the limited sample size and the heterogeneous distribution of subgroups according to drug types may have reduced the statistical power of some comparisons. This limitation is also related to the specific nature of the study population, as pediatric liver transplant recipients represent a highly specialized and relatively small clinical group. Another limitation of this study is that only a limited number of biochemical parameters were evaluated. Future studies including a broader range of liver-related biomarkers may provide a more comprehensive understanding of the relationship between physiological and psychological factors.

## 5. Conclusions

In conclusion, this study showed that depression levels were generally low in clinically stable pediatric liver transplant recipients. A significant positive relationship was identified between post-traumatic stress and depression, indicating that higher PTSD levels were associated with higher depression levels. Although a modest negative correlation was observed between bilirubin and depression scores, biochemical indicators did not demonstrate consistent associations with psychological symptoms overall. Additionally, no significant differences in depression levels were found according to sociodemographic and transplant-related characteristics. These findings suggest that post-transplant follow-up should not rely solely on biological parameters and that regular psychological assessment, particularly trauma-focused evaluation, may contribute to improved clinical outcomes.

## Figures and Tables

**Table 1:** Age-related variables and scale averages for transplanted children (N =5 0).

Variables	Min	Max	*X* ± SD	Md	Q1–Q3
Age	8	18	13.10 ± 2.97	13.50	11.00–15.25
Transplant age (months)	5	204	78.82 ± 58.34	66.00	24.00–132.00
Mother’s age	28	53	38.32 ± 5.86	38.50	34.00–41.25
Father’s age	29	55	41.94 ± 6.16	42.00	38.00–46.00
CRIES	0	43	12.42 ± 12.38	8.00	2.75–20.00
PHQ-9	0	14	3.68 ± 4.43	1.00	0.00–8.25
AST	12	105		32.00	24.75–58.50
ALT	9	255		27.00	16.00–71.00
Bilirubin	0.26	22.26		0.59	0.41–0.89
GGT	9	331		27.50	17.00–93.50
BMI	10.70	31.20		19.10	16.55–21.72

Min: Minimum, Max: Maximum, X ± SD: Mean ± Standart Deviation, Md: Median, CRIES: Child Revised Impact of Event Scale, PHQ-9: Patient Health Questionnaire–Depression, Continuous variables are presented as median (Q1–Q3) due to non-normal distribution. Reference ranges: AST = 10–40, ALT = 5–35, Bilirubin = 0.2–1.2, GGT = 5–25, BMI = 18.5–24.9.

**Table 2:** Comparison of depression levels among transplanted children according to certain socio-demographic characteristics and transplant-related characteristics (N = 50).

Variables				Patient Health Questionnaire–Depression (PHQ-9)			
		n	%	*X* ± SD	Median (Q1–Q3)	t/U/X^2^	*p*
Gender *	Male	26	52.0	3.57 ± 3.91	2.00 (0–7.50)	−0.168	0.868
	Female	24	48.0	3.79 ± 5.03	0.50 (0–8.75)		
Continuing education *	No	8	16.0	4.62 ± 5.73	2.50 (0–10.50)	0.529	0.611
	Yes	42	84.0	3.50 ± 4.20	1.00 (0–8.25)		
Chronic illness in the family *	No	40	80.0	3.37 ± 4.25	0.00 (0–6.50)	−0.862	0.405
	Yes	10	20.0	4.90 ± 5.17	4.00 (0–9.25)		
Type of transplantation **	Cadaver	6	12.0	4.66 ± 4.27	4.00 (0.75–9.25)	99.500	0.569
	Living donor	44	88.0	3.54 ± 4.49	1.00 (0–7.75		
Immunosuppressive medication *	Takrolimus	43	86.0	3.44 ± 4.34	1.00 (0–8)	−0.837	0.428
	Everolimus	7	14.0	5.14 ± 5.08	5.00 (1–9)		
Presence of additional diseases *	No	49	98.0	3.57 ± 4.41	1.00 (0–7.50)	−1.217	0.230
	Yes	1	2.0	9.00 ± 0.00	9.00 (9–9)		
Mother’s education level ***	None	17	34.0	2.58 ± 4.45	0.00 (0–4)	2.409	0.492
	Elementary school	24	48.0	4.16 ± 4.54	2.50 (0–8.75)		
	High school	5	10.0	4.20 ± 4.08	5.00 (0–8)		
	University	4	8.0	4.75 ± 4.92	5.00 (0.25–9)		
Father’s education level ***	None	8	16.0	3.00 ± 5.42	0.00 (0–7)	2.877	0.411
	Elementary school	30	60.0	3.20 ± 4.08	1.00 (0–5.25)		
	High school	8	16.0	5.87 ± 4.85	7.00 (0.5–9)		
	University	4	8.0	4.25 ± 4.42	4.00 (0.25–8.50)		

* Independent groups *t*-test, ** Mann Whitney U, *** Kruskal Wallis H (X2), n: Frequency, %: Percentage, Q1 = 25th percentile (first quartile), Q3 = 75th percentile (third quartile).

**Table 3:** Correlation between CRIES scores of transplanted children, age-related variables, and liver enzyme levels with PHQ-9 scores (N = 50).

		Age	Transplant Age	BMI	AST	ALT	Bilirubin	GGT	CRIES
**PHQ-9**	**r**	−0.268	0.223	−0.027	−0.124	−0.062	−0.310 *	−0.133	0.414 **
	***p***	0.059	0.119	0.853	0.391	0.671	**0.028**	0.356	**0.003**

* Correlation is significant at the 0.05 level, ** Correlation is significant at the 0.01 level, r: Pearman’s Correlation Coefficient, CRIES: Child Revised Impact of Event Scale, PHQ-9: Patient Health Questionnaire–Depression.

**Table 4:** Effect of the CRIES total score of children who have undergone transplantation on the PHQ-9 total score.

Model	Patient Health Questionnaire–Depression (PHQ-9)					95.0% CI	
	B	SE	Beta	t	*p*	Lower Bound	Upper Bound
(Constant)	2.336	1.638	-	1.426	0.161	−0.965	5.637
AST	0.045	0.060	0.258	0.758	0.453	−0.075	0.165
ALT	−0.016	0.025	−0.176	−0.634	0.530	−0.067	0.035
Bilirubin	−0.381	0.208	−0.283	−1.835	0.073	−0.800	0.038
GGT	−0.012	0.012	−0.216	−1.031	0.308	−0.036	0.012
CRIES	0.117	0.054	0.326	**2.149**	0.037 *	0.007	0.226
Durbin Watson: 2.383 R: 0.380 ^a^ R^2^: 0.144 F: 1.482 *p*: 0.215 ^a^							

* *p* < 0.05; Regression Analysis (F); Coefficient Analysis (t); Summary statistics Regression Coefficient (SE: Standard Error) is given as a value, CRIES: Child Revised Impact of Event Scale. ^a^ Overall model statistics.

## Data Availability

The data supporting the findings of this study are available from the corresponding author upon reasonable request. The data are not publicly available due to privacy and ethical restrictions.
